# Feasibility of Home-Based Transcranial Direct Current Stimulation with Telerehabilitation in Primary Progressive Aphasia—A Case Series

**DOI:** 10.3390/brainsci15070742

**Published:** 2025-07-10

**Authors:** Anna Uta Rysop, Tanja Grewe, Caterina Breitenstein, Ferdinand Binkofski, Mandy Roheger, Nina Unger, Agnes Flöel, Marcus Meinzer

**Affiliations:** 1 Department of Neurology, University Medicine Greifswald, 17475 Greifswald, Germany; anna.rysop@med.uni-greifswald.de (A.U.R.); nina.unger@med.uni-greifswald.de (N.U.); agnes.floeel@med.uni-greifswald.de (A.F.); 2 Department of Technology and Health for People, Jade University of Applied Sciences, 26389 Oldenburg, Germany; tanja.grewe@jade-hs.de; 3 Department of Neurology, University of Münster, 48149 Münster, Germany; breitens@uni-muenster.de; 4 Division for Clinical Cognitive Sciences, Department of Neurology, University Hospital, RWTH Aachen, 52074 Aachen, Germany; fbinkofski@ukaachen.de; 5 Institute for Neuroscience and Medicine (INM-4), Research Center Jülich, 52425 Jülich, Germany; 6 Department of Psychology, Carl von Ossietzky University Oldenburg, 26129 Oldenburg, Germany; mandy.roheger@uni-oldenburg.de; 7 German Centre for Neurodegenerative Diseases (DZNE), 17487 Greifswald, Germany

**Keywords:** primary progressive aphasia, telerehabilitation, home-based transcranial direct current stimulation, feasibility, tolerability, safety

## Abstract

**Background:** Primary progressive aphasia (PPA) is a neurodegenerative disease characterised by progressive impairment of speech and language abilities. Intensive speech and language teletherapy combined with remotely supervised, self-administered transcranial direct current stimulation (tDCS) may be suited to remove barriers to accessing potentially effective treatments, but there is only limited evidence on the feasibility of this combined approach. **Methods:** This pilot case series investigated the feasibility, tolerability and preliminary efficacy of a novel telerehabilitation programme combined with home-based, self-administered tDCS for people with primary progressive aphasia (pwPPA). The intervention programme was co-developed with pwPPA and their caregivers, to reflect their priorities regarding treatment content and outcomes (i.e., naming, functional communication). **Results:** Two pwPPA successfully completed the telerehabilitation intervention with daily naming training and communicative-pragmatic therapy paired with tDCS, over 10 consecutive workdays. Caregivers assisted in the setup of equipment required for teletherapy and home-based tDCS. Participants successfully completed the programme with a 95% completion rate. Home-based tDCS was well tolerated. Both participants showed improvements in naming and communication, suggesting preliminary efficacy of the intervention. **Conclusions:** Overall, this study demonstrates the feasibility and potential benefit of a novel, easily accessible and patient-relevant telerehabilitation intervention for pwPPA, which requires confirmation in a future larger-scale exploratory trial.

## 1. Introduction

Primary progressive aphasia (PPA) is a neurodegenerative disease that predominantly affects speech and language abilities [1]. Based on the consensus criteria of Gorno-Tempini and colleagues [2], PPA is typically classified into three variants (semantic variant, logopenic variant and nonfluent-agrammatic variant of PPA). These variants differ regarding speech and language symptoms, with word-finding difficulties being a frequent and early symptom in all variants [3,4]. Currently, no pharmacological interventions are available for improving or delaying the progression of the disease [5]. However, speech and language therapy (SLT) can have positive effects on speech and language function in people with PPA (pwPPA), their quality of life and societal participation [4,6,7]. Intervention studies typically target word-finding difficulties with lexical or phonological naming interventions [6,8] and have shown positive effects, especially on trained words [7]. In addition, functional communication approaches, such as script training [8] or discourse treatments [9] have been used to improve everyday communication [7].

Recently, there has also been growing interest in the use of non-invasive brain stimulation, such as transcranial direct current stimulation (tDCS), to enhance and extend the positive effect of SLT in pwPPA. TDCS is a non-invasive brain stimulation method that uses weak electrical currents, applied via scalp-attached electrodes, to modulate cortical excitability and plasticity in underlying brain regions [10]. Cortical excitability can be either increased (e.g., anodal tDCS) or decreased (i.e., cathodal tDCS), depending on the polarity of the induced current. In neurodegenerative diseases such as PPA, the rationale for pairing anodal tDCS with a behavioural intervention is mainly to increase neuroplasticity in the remaining language network [11,12]. TDCS is considered to be safe, low-cost and easy to administer, making it a promising adjunct to behavioural therapy for stroke-based aphasia [13]. However, there is currently only limited evidence for add-on effects of tDCS when combined with SLT in PPA [14]. Indeed, only a small number of statistically underpowered, randomised controlled trials have explored the add-on effects of tDCS administered to frontal or temporal cortices in combination with SLT (e.g., picture naming training or Constraint-Induced Aphasia Therapy) on language performance in PPA. These studies provided preliminary evidence for the potential beneficial effects of tDCS during naming tasks, especially for trained words [14,15]. However, such combined treatments are currently only available in specialised centres (e.g., university hospitals). Furthermore, these combined interventions need to be administered frequently (i.e., several times per week) over several weeks to be most effective [14,15,16]. This places high demands on pwPPA and their carers in terms of time, mobility and mobility-related finances. Home-based, remotely supervised but self-administered applications of tDCS have recently become a research focus [17,18,19], to facilitate access to potentially effective treatment. Such approaches can be combined with behavioural interventions delivered as teletherapy. Indeed, several studies have shown that online-delivered telerehabilitation might be as effective as in-person treatments [20,21,22], which is also supported by two recent reviews [23,24]. For example, one study showed that internet-based SLT services are technologically feasible and can be used successfully by pwPPA. In addition, eight personalised internet-based SLT sessions (including impairment-, activity- and/or participation-based approaches and disease education) resulted in improved functional communication and communication confidence [25]. Another study directly compared the efficacy of treatment delivered in-person with teletherapy in pwPPA and reported similar effect sizes for both delivery modes [20]. These results laid the groundwork for the development of novel treatment approaches in a home-based setting, such as combining home-based behavioural interventions with self-administered tDCS.

Nonetheless, home-based interventions require a high level of technical skills, both in the handling of the stimulation devices and participation in online-delivered SLT. To ensure that pwPPA can participate in a treatment that requires a technically demanding setup, feasibility needs to be evaluated in the target population. To the best of our knowledge, only two other studies so far have investigated the feasibility of home-based tDCS in combination with telerehabilitation in small samples of pwPPA [26,27]. A recent open-label observational study combined an individualised, remotely delivered naming treatment with home-based anodal tDCS over the left inferior frontal gyrus [27]. After 20 sessions spread over four weeks, ten pwPPA showed significantly improved naming ability, with more pronounced gains for trained compared to untrained words. Importantly, all participants completed the intervention, demonstrating the high feasibility of the combined home-based tDCS and SLT approach. In another pilot study, Neophytou and colleagues [26] combined home-based tDCS with a word repetition treatment targeting verbal short-term memory and working memory. Seven pwPPA received anodal or sham tDCS over the left supramarginal gyrus (SMG) in a cross-over design with ten sessions. The results of this pilot study indicated that home-based tDCS is feasible for pwPPA. Anodal tDCS over the left SMG coupled with word repetition training improved verbal short-term memory (measured as word span backward) and generalised to other language functions (spelling of real and pseudowords; verbal learning: retention and delayed recall; [26]). However, this treatment targeted verbal short-term memory and working memory, i.e., functional domains that may not have the highest priority for pwPPA.

Indeed, a recent international consensus-based approach identified the top five priorities for the research and treatment of pwPPA and their caregivers [28]. The highest ranked outcomes included to “participate in conversations with family and friends”, to “get words out” and to “convey a message by any means” [28]. These findings suggest that the sole use of naming treatments is insufficient to meet the needs of pwPPA and their caregivers. This finding is echoed by the results of a qualitative study conducted by our group, which used semi-structured interviews to identify the needs and priorities of pwPPA and caregivers with respect to SLT [29]. Here, naming and communication therapy were considered equally important by both stakeholder groups. In addition, we assessed technological barriers and possible solutions for pwPPA. Based on the results of this co-design phase, we adapted an established treatment comprising both naming training [30] and communicative-pragmatic intervention [31] for teletherapy use in pwPPA. We also involved pwPPA and caregivers in the development and testing of a detailed step-by-step manual and corresponding training for combined speech-language teletherapy and home-based tDCS [29].

**Figure 1:**
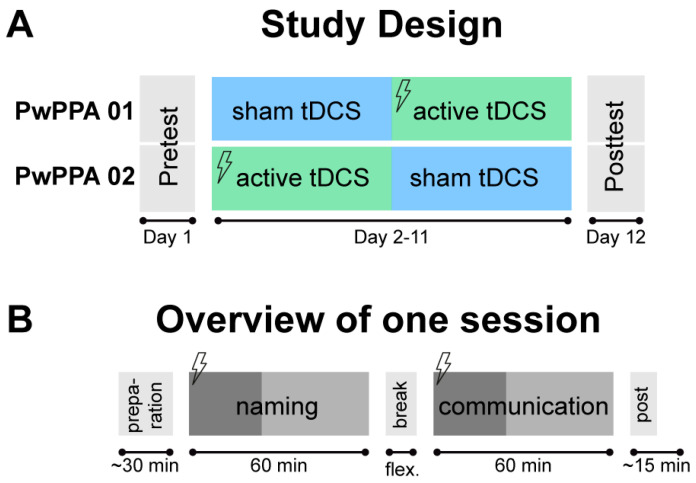
Study design. (**A**) Overview of the study design for both participants. Both participants received active and sham tDCS in a cross-over design, with counterbalanced order. (**B**) Overview of an exemplary intervention session. Flashes indicate transcranial direct current stimulation that was conducted during the first 20 min (dark grey bars) of each SLT session, respectively; flex = flexible duration of breaks.

In the present case series, we aimed to demonstrate the feasibility and tolerability of this novel home-based telerehabilitation programme that was co-designed together with primary stakeholders. The telerehabilitation programme combines synchronous speech-language teletherapy with self- or caregiver-administered home-based anodal tDCS over ten consecutive days (two weeks). After an initial on-site training session (supported by patient-tailored training manuals (available via [29]), a usability test and competency checklists), two people with PPA completed an online-delivered SLT, involving word level [30] and communicative-pragmatic therapy [31]. Anodal tDCS was administered in a cross-over design (i.e., one week of active tDCS and one week of sham tDCS; see Figure 1). This approach allowed for the investigation of tolerability and acceptability of active vs. sham stimulation in the same person. Caregivers supported the participants with the setup of the brain stimulation equipment and the laptop used for telerehabilitation. In addition, one researcher was present during all sessions to monitor the feasibility of the approach. Naming and communicative-pragmatic performances were assessed before and after treatment.

## 2. Materials and Methods

The reporting of the materials and methods sections follows the CONSORT extension for Pilot and Feasibility Trials [32].

### 2.1. Trial Design

The study included 12 sessions and was conducted in a home-based setting (i.e., at the participants’ homes; see Figure 1A for an overview of the study design). The first day comprised a baseline session in which language assessments (see below for details) were administered in-person by one researcher. Additionally, the participants received training for the use of the teletherapy platform and tDCS on the same day. The baseline session was followed by 10 therapy days spread across two weeks, in which participants received two SLT sessions per day via a teleconferencing platform, in addition to self-applied active or sham tDCS (see below for a detailed description of the intervention). Each of the ten therapy days consisted of the fixed sequence of one hour of naming training followed by one hour of communicative pragmatic therapy (see Figure 1B). There was a break with a flexible duration between these therapies, ranging from 8 to 31 min (mean duration: 15 min). Each therapy component was combined with tDCS, hence each pwPPA received tDCS twice per day. The stimulation duration was set to 20 min and started in parallel with SLT. Therapy continued after the stimulation was ramped down, exploiting known after-effects of tDCS that outlast the actual stimulation duration [33]. All equipment was provided for the duration of the study participation. Aside from the participants, two people were involved as follows: one on-site investigator and one remote speech and language therapist. The investigator was present during the whole time at the participants’ homes to monitor and document all procedures, and to conduct the baseline and post-test assessments. This approach was chosen for the present pre-trial pilot feasibility study to document in detail potential problems that may arise when using home-based tDCS combined with teletherapy. Teletherapy was delivered by a trained speech-and-language therapist using the secure video conferencing platform BigBlueButton (https://bigbluebutton.org/). The speech and language therapist had experience with delivering teletherapy but neither involving pwPPA, nor the specific interventions used in the present study. The therapist was trained in the interventions before participants were enrolled. After the intervention phase, a post-test session was conducted, including the same language assessments as presented at the baseline assessment.

### 2.2. Participants

Participants were recruited via convenience sampling from a pool of participants, who had already taken part in other studies at the University Medicine Greifswald, including earlier phases of this project and had agreed to be re-contacted for future studies. Participants were eligible if they had a neurologist-confirmed diagnosis of PPA (each variant possible) and if they were adults (at least 18 years old), German (native) speakers, because the speech and language programme is currently only available in German. In addition, the availability of a caregiver during the treatment period was required to provide assistance with the technical setup (i.e., teletherapy, tDCS). Exclusion criteria comprised contraindications for receiving tDCS, such as cardiac pacemakers or cranial implants [34], or a history of other neurological conditions (e.g., stroke, epilepsy).

Two pwPPA participated in this feasibility case series together with a caregiver (spouse). Participant 1 was a 59-year-old woman, with a neurologist-confirmed diagnosis of logopenic variant of PPA. She had received the diagnosis less than a year prior to participation. Participant 2 was a 68-year-old man. He had been diagnosed by a neurologist with the logopenic variant of PPA for approximately one year. Both participants attended with their spouses, with whom they live together.

All participants (pwPPA and caregivers) provided written informed consent prior to participation and received financial reimbursement for study participation. The study was performed in accordance with the guidelines of the Declaration of Helsinki and approved by the local ethics committee at the University Medicine Greifswald (BB 196/23).

### 2.3. Assessments

In the baseline naming assessment 336 photographs of objects (nouns) were presented via a custom computerised programme developed by our group [30,35]. All words were presented for a maximum duration of 20 s, with an automatic break after 40 items. Responses were rated by the investigator via button press as correct or incorrect. Words that could not be named after 20 s were automatically rated as incorrect. Each word was repeated twice in a randomised sequence during the assessment. A total of 60 words that could not be named in at least one of two instances were randomly selected as training items for the subsequent naming training. The rest of the items remained untrained.

To determine the focus of the communicative-pragmatic therapy, we used the German communicative-pragmatic screening KOPS [36]. Briefly explained, KOPS assesses verbal and non-verbal communication skills through nine subtests of increasing difficulty and complexity. These subtests include: (1) Understanding words in context, (2) Referring to everyday objects, (3) Basic communication (agreement/disagreement), (4) Conveying personal information, (5) Conveying general information, (6) Complex communicative actions (individual actions), (7) Complex communicative actions (role-plays), (8) Understanding and reconstructing directions and (9) Generating directions. For therapy, two subtests were selected in which participants scored less than 80% correct. In the case of multiple subtests scoring less than 80% correct, we deviated from the KOPS manual recommendation and selected the two subtests that were most relevant to the participants’ daily lives. Those two subtests were the initial subtests for the communicative-pragmatic therapy. Note that this therapy programme is designed in a way that allows the progression to other subtests when a certain performance level is reached (see below for more details on therapy progression). For this reason, it is possible that more than two subtests can be treated over the course of 10 days of therapy. Both assessments were conducted in person by one investigator.

### 2.4. Interventions

The speech and language intervention comprised an established computerised naming training [30,35] and communicative-pragmatic therapy (ESKOPA-TM; [31]).

The computerised and repetitive naming training was delivered for approximately one hour daily during the two-week intervention phase. A subset of 60 words that could not be named twice during repeated baseline assessments were trained using the vanishing cues method [37], with auditory and graphemic cues at four hierarchical levels of difficulty [38]. The hierarchy of cues was set as follows. At the first level, the maximum number of cues was available, as the picture was paired with the full spoken and written form of the target word. In the remaining levels, the cues were gradually reduced. At the second level, the written word form disappeared, and auditory cues were reduced to the first two phonemes and subsequently at the third level to the first phoneme. Lastly, at the fourth level of difficulty, the picture was presented without any accompanying cues. PwPPA were instructed to name the picture that was presented to them on the screen across all levels of difficulty. Difficulty levels were adapted based on the participants’ performance. To progress to the next difficulty level, participants had to name over 80% correct in one level. Otherwise, the difficulty level was reduced, i.e., the words were presented with more cues in the following. The items remained on the screen for a maximum of 20 s. Training items were presented via screensharing, and the speech and language therapist rated the performance as correct or incorrect via button press on the keyboard.

In the communicative-pragmatic therapy block [31], participants were trained on two of the tasks on which they had scored less than 80% correct in the communicative-pragmatic baseline assessment (KOPS). Each session had a duration of one hour and each task was trained for approximately 30 min. For example, if the task types (6) “complex communicative actions (individual actions)” and (7) “complex communicative actions (role-plays)” were selected for one participant, individual communicative actions were trained in the first half of the session and communicative role-plays were trained in the second half of the session. The task type “complex communicative actions (individual actions)” consisted of scenarios in which participants had to solve a communicative task in the form of an individual communicative action. In contrast, in the task type “complex communicative actions (role-plays)” participants had to solve a communicative task in the form of a dialogue with a sometimes difficult and/or impolite communication partner (speech and language therapist). Stimuli or scenarios were individualised based on participants’ communication needs (e.g., personally relevant dialogues or topics). At the beginning of each task, monitoring items were used to assess the participants’ progress on the respective task. If they were able to solve all monitoring items on two consecutive days, participants could progress to the next task. Otherwise, they remained at the same task. Monitoring results and therapy contents were documented by the speech-language therapist. When needed, therapy items or maps were shared using the screensharing function of BigBlueButton. For some tasks, the interactive whiteboard of BigBlueButton was used.

### 2.5. Transcranial Direct Current Stimulation (tDCS)

TDCS was delivered via the DC-Stimulator Mobile (NeuroConn), a device that has specifically been designed for home-based applications. Electrodes were placed in an elastic knitted cap with integrated 5 × 5 cm electrodes, recently developed for home-based tDCS to enable easy, precise and reproducible positioning of the electrodes (https://www.neurocaregroup.com/technology/tes-cap, accessed on 8 July 2025). Cap size was based on individual head height (defined as the distance between the corner of the eye and the highest point of the head) and circumference to ensure a good fit. For this feasibility trial, we chose a bilateral prefrontal montage as previously suggested [12], and the anode was placed over F3 of the 10–20 electrode positioning system. F3 corresponds to the left dorsolateral prefrontal cortex. The cathode was placed contralaterally over F4. The stimulation protocol was pre-configured by NeuroConn and saved on two storage modules (i.e., one for the sham stimulation protocol and one for the active stimulation protocol). During active stimulation, participants received 20 min of 1.0 mA, during sham stimulation the current was ramped up for 30 s in the beginning to induce similar sensations as compared to active stimulation. Participants were instructed to use one storage module per week. Each storage module contained a maximum of 14 stimulation sessions. Ten stimulation sessions per storage module were required for the treatment, the remaining four served as back-up in case a stimulation session was accidentally terminated directly after it was started by pushing the starting button twice. Caregivers assisted with the tDCS setup before and after the therapy but were not present during therapy. In particular, caregiver assistance was needed for the handling of the cap, to ensure correct placement and to fill in the electrodes with saline solution (0.9% NaCl). Correct cap placement was determined through visual inspection by the patient, caregiver and the remote speech-language therapist via videoconferencing and additionally confirmed by the on-site researcher.

### 2.6. Randomisation and Blinding

Both participants received active and sham tDCS in a cross-over design. The order of the tDCS conditions was counterbalanced. One participant received active tDCS in the first week, followed by sham tDCS in the second week. The order was reversed for the other participant. Participants, caregivers and the speech-language therapist were blinded with respect to the stimulation condition. For safety reasons, the investigator that was present at the participants’ homes was unblinded. Due to relatively high pain ratings during sham tDCS (participant 2, week one), the therapist was unblinded in the second week (for details see results section).

### 2.7. Question, Hypotheses and Aims

The case study presented here aimed to investigate the following research question: Is the combination of self- or caregiver-administered combined tDCS-SLT telerehabilitation feasible and tolerable? Thus, the main goal was to assess the feasibility and tolerability of the home-based implementation of tDCS and speech-language teletherapy rather than treatment efficacy. However, we also assessed naming and communicative-pragmatic performance before and after the intervention to capture preliminary intervention-related training effects, which could serve as the basis for the sample estimation of a larger Phase IIb interventional trial.

### 2.8. Data Analysis

The feasibility of the entire procedure was defined by the number of actual as compared to a priori planned therapy sessions. A minimum of 80% of sessions had to be completed to be considered feasible. In addition, we assessed the feasibility of tDCS by the number of completed stimulations (20 in total). Likewise, a minimum of 80% of simulations had to be completed to be considered feasible. Finally, we calculated the percentage of actions that were carried out independently by patient–caregiver dyads using a competency checklist. The competency checklists included eleven tDCS-related and four teletherapy-related actions that patient–caregiver dyads learned during the training and that could be performed by either the patient, the caregiver or both together. With this procedure, we aimed to document the independence of the patient–caregiver dyads. Proportions of independently executed actions were calculated across all actions and for each therapy day. Competency checklists were adapted from [18,39] and extended for the purpose of our study (see [29] for a detailed description of the adaptation and development of competency checklists). To assess the tolerability and safety of stimulation, we used the tDCS adverse events rating scale proposed by Antal and colleagues [34] and the Wong-Baker FACES Pain Rating Scale (https://wongbakerfaces.org/). The latter is a visual and numerical rating scale on which participants can indicate their perceived discomfort or pain on a scale from zero (no discomfort/pain) to ten (high discomfort/pain). Both rating scales were administered each day after completion of both stimulation sessions. Finally, to assess the efficacy of the SLT, we investigated the changes in naming performance using a custom computerised naming assessment (pre-post change in naming score for trained and untrained words, separately). The pre-post change in communicative-pragmatic functions was assessed using the KOPS and compared descriptively. Performance differences in the naming assessment for trained and untrained object names were compared using the McNemar test [40]. Data were analysed and visualised using R [41].

## 3. Results

### 3.1. Feasibility

Feasibility was assessed for the combined home-based tDCS-behavioural intervention. Both participants completed all sessions (100% intervention completion rate) indicating a high level of acceptability for the whole intervention. With regard to home-based tDCS, both participants were able to commence all stimulation sessions. Participant 2 terminated one of 20 stimulations after 15 min (i.e., five minutes earlier) due to mild headaches and major fatigue (95% stimulation completion rate). From a technical perspective, no challenges occurred with respect to the brain stimulation equipment and handling (i.e., all participants were able to prepare and administer the brain stimulation). However, a number of technical challenges were encountered, most of which were related to a poor internet connection. Participant 1 had no internet connection on 2/10 days, and partial internet outage on one additional day. On these days, teletherapy was continued by connecting the computer via a mobile hotspot to the internet. Poor internet connection also affected the computerised naming training on four days, resulting in slow and sometimes asynchronous presentation of image and sound.

**Figure 2:**
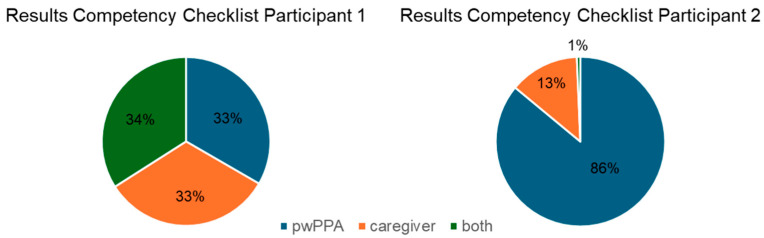
Results of the competency checklists showing the percentage of independently executed actions by pwPPA (blue), caregivers (orange) or both (green), averaged across all intervention sessions. Both participants differed substantially in their independence in the preparatory actions required for the home-based tDCS and teletherapy setup.

Participants differed in their independence during the setup of the combined tDCS-SLT teletherapy approach (Figure 2). Participant 1 relied heavily on their caregiver during the tDCS administration. With regard to the laptop and the telerehabilitation platform, this participant needed considerable caregiver support including all preparatory steps (e.g., entering the password and starting the teleconferencing platform). Despite prior training, the participant was unable to use the whiteboard independently. Therefore, substantial caregiver assistance was needed here as well. In contrast, participant 2 performed all actions related to the brain stimulation independently, with the exception of filling the electrodes with saline. Due to a high level of technical skills, participant 2 was able to perform all actions related to telerehabilitation independently. Caregiver assistance was only needed to fill in the electrodes with the saline solution.

### 3.2. Tolerability and Safety

**Figure 3:**
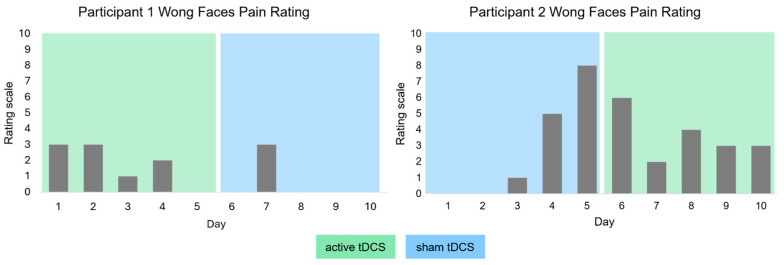
Results of the Wong Faces pain rating scale. Green shaded area marks the time period in which active tDCS was applied, blue indicates sham tDCS. Higher values indicate higher pain ratings.

Tolerability and safety were assessed using an adverse events rating scale and pain rating scale. Overall, no serious adverse events occurred. Participant 1 rated the perceived sensations as mild (maximum pain rating 3/10; see Figure 3 left). The following sensations were reported: Tingling (mild on 2/10 days, none on the remaining days), Pain (0/10 days), Burning (moderate on 1/10 days, mild on 1/10 days, none on the remaining days), Warmth/Heat (mild on 1/10 days, none on the remaining days), Metallic/Iron taste (0/10 days), Fatigue/decreased alertness (0/10 days). If reported, the sensations were always perceived in the beginning of the stimulation and were present only initially. Participant 1 indicated the sensations were mostly located close to the electrodes or diffuse on one day. Self-reports indicated that these sensations did not affect participant 1 during the intervention or in her general state.

Participant 2 rated the perceived sensations as mild to moderate on most days and one time as painful (maximum pain rating 8/10; see Figure 3 right). The following sensations were perceived: Tingling (mild on 2/10 days, none on the remaining days), Pain (strong on 2/10 days, mild on 5/10 days, none on 3/10 days), Burning (strong on 3/10 days, moderate on 1/10 days, mild on 2/10 days, none on 4/10 days), Warmth/Heat (strong on 1/10 days, moderate on 1/10 days, mild on 3/10 days, none on the remaining days), Metallic/Iron taste (0/10 days), Fatigue/decreased alertness (strong on 1/10 days, mild on 2/10 days, none on the remaining days). Perceived sensations occurred in the beginning of the stimulation on most days and towards the end of the stimulation on one day. These sensations did not last until the end of the session; they were evident only initially or persisted approximately until the middle of the session. When these sensations were strong, participant 2 indicated that they affected his general state considerably. Participant 2 indicated the sensations were mostly located close to the electrodes and under/between the electrodes. Notably, pain was most pronounced during sham tDCS (see Figure 3) and decreased again during active tDCS, likely due to an adjustment of the instructions on how to fill the electrodes with saline solution from day 6 onwards. In addition to filling the electrodes via the opening in the sponge electrode, the electrode was carefully moistened with saline solution from the inside of the cap, to ensure better conductivity and less pronounced adverse events.

In the final debriefing, both participants were unable to identify in which of the two weeks the active stimulation had been administered.

### 3.3. Speech and Language Performance

**Figure 4:**
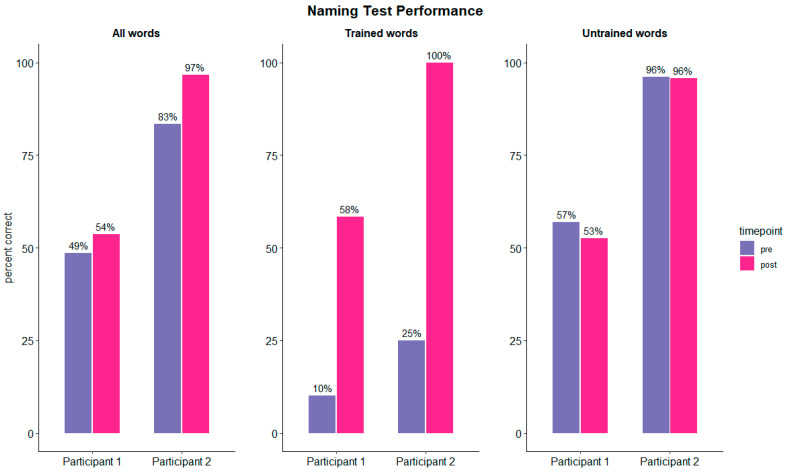
Naming results pooled across tDCS conditions. The left panel displays the performance in the naming task for all words (trained and untrained). The middle panel displays naming performance of trained words. The naming performance of untrained words is shown in the right panel.

Both participants showed improvement in their overall naming performance after the intervention compared to the baseline assessment (Figure 4, left panel). Naming improvement was highly significant for trained words in both patients, indicating a large training effect (participant 1: Χ^2^ = 52.4, *p* < 0.001, Cohen’s g = 0.47, large effect; participant 2: Χ^2^ = 88.01, *p* < 0.001, Cohen’s g = 0.5, large effect). Improvement was restricted to trained items (Figure 4, middle panel), naming performance of untrained items slightly decreased or remained the same (participant 1: Χ^2^ = 3.39, *p* = 0.07, Cohen’s g = 0.08; participant 2: Χ^2^ = 0.03, *p* = 0.87, Cohen’s g = 0.03) (Figure 3, right panel).

Next, we were interested in the performance change in communicative-pragmatic abilities, measured by the assessment tool KOPS. Following the predefined rules of progression and regression, Participant 1 received training in three subtests of the communicative-pragmatic training (six times Easy dialogues, four times Referring to everyday objects and ten times Complex communicative actions (role-plays)). Participant 2 received treatment of four subtasks (Complex communicative actions (individual actions), Understanding/reconstructing directions, Generating directions, for five sessions each). Both participants showed improvement in their communicative pragmatic skills, indicated by a change in the total KOPS score (Table 1 bold), that was driven by the trained subtests (Table 1, italics). Subtests that were trained during the intervention period showed improvement in both participants, except for the subtest “Referring to everyday objects” in Participant 1 (Table 1; please see Section 4.3. for further discussion). Similarly to the outcomes of the naming intervention, this indicates a considerable training effect.

## 4. Discussion

In the present pilot study, we investigated the feasibility, tolerability, safety and preliminary efficacy of a novel home-based tDCS and telerehabilitation SLT programme in two people with PPA. Our main findings were as follows: First, both participants were able to complete all intervention and stimulation sessions with support of their caregivers, indicating high feasibility of the intervention programme. Second, tDCS was generally well tolerated, even though adjustments to the amount of saline solution were required in one patient to improve tolerability. There were no treatment-limiting serious adverse events. Lastly, improved naming and communication was observed in both patients, providing preliminary evidence for the efficacy of our novel approach. Overall, these results demonstrate the feasibility, tolerability, safety and preliminary efficacy of the novel combination of naming and communicative-pragmatic teletherapy with home-based, self- or caregiver-administered tDCS in two people with PPA.

### 4.1. Telerehabilitation Combined with Home-Based tDCS Is Feasible for People with PPA

A total of 19 out of 20 intervention sessions were completed by both patient–caregiver dyads. This high completion rate indicates that the combination of self-administered and caregiver-supported home-based tDCS with speech-language teletherapy is feasible and acceptable for pwPPA. These findings are in line with the results of two previous pilot studies demonstrating feasibility of home-based tDCS in small groups of pwPPA (N = 7, [26]; N = 10, [27]) and also those of a recent case report that included a patient with the behavioural variant of frontotemporal dementia [42]. Importantly, our results extend the findings of these studies by demonstrating the feasibility of administering two stimulation sessions per day. In addition, by using the competency checklists introduced by Rysop and colleagues [29], we were able to investigate the degree of independence in a more detailed manner. Although both participants had the same PPA variant and similar disease duration, their competence profiles differed substantially. Therefore, the level of support needed from caregivers may depend on several factors, including the severity of the disease and premorbid technical skills. Notably, a recent study that investigated feasibility of home-based tDCS in ten pwPPA [27] reported that 70% of participants required assistance by a caregiver. Another study suggested that experience with computers and support from caregivers are critical for teletherapy success [25]. This may be specifically relevant regarding unforeseen technological challenges (e.g., a poor internet connection) requiring high problem-solving abilities, which can be reduced in pwPPA [22,29]. In line with our findings, these results underscore the necessity of including a caregiver or support person when planning home-based tDCS trials for people with PPA.

### 4.2. Home-Based Self- or Caregiver-Administered tDCS Is Tolerable for People with PPA

In the present study, no serious adverse events were reported, indicating that home-based tDCS was well tolerated. This is further supported by the high completion rate of tDCS sessions (95%). Both participants reported mild tingling and mild burning sensations at the beginning of some stimulation sessions and one participant additionally reported a feeling of fatigue during the stimulation. Overall, the occurrence of mild adverse events is in line with other interventional tDCS studies in PPA (for review see [14]), and also with tDCS studies in other populations [34]. With regard to home-based tDCS in pwPPA, one pilot study reported mild adverse events, including mild tingling and warmth in the beginning of stimulation sessions [27] while another study only stated that tDCS was well tolerated with initial tingling or itching in some participants [26]. However, none of these studies reported detailed pain or adverse event ratings. To maximise safety and to gain information on the tolerability of tDCS, especially in home-based settings, it is essential to continuously monitor and document adverse events across the intervention period. In the current study, one participant reported relatively high pain ratings during the sham condition, when the electric current was administered for only 30 s to mimic the sensations of active tDCS. Nonetheless, only one of 20 stimulation sessions was terminated by the patient and the optimisation of saline injection into the electrode pads (i.e., not only from the outside, but also from inside the cap), substantially decreased pain ratings. Hence, this outcome provided valuable information that the default way of applying saline solution suggested by the manufacturer may induce adverse effects even during sham tDCS and a possible solution to improve tolerability. Moreover, individuals react differently to active tDCS stimulation, and some people might be more susceptible to pain. Therefore, to maximise adherence and to exclude participants who may find the active stimulation too uncomfortable or even painful, we recommend including a “test stimulation” in the training phase prior to the actual intervention. Such procedures have already been proposed in other diseases (e.g., [43] for people with Parkinson’s disease). With respect to participant blinding, (1) both participants reported perceiving sensations also in the sham condition and (2) neither pwPPA, nor the caregivers were able to identify the stimulation order in the final debriefing. This suggests effective blinding with a new cap solution that was specifically developed for this study. Effective blinding using similar approaches is also consistent with previous studies on home-based tDCS applications in pwPPA [26,27].

In the current study, we conducted baseline assessments and training in teletherapy and tDCS handling on the same day, because participants had already participated in usability tests [29] and were therefore familiar with the procedures. Hence, only a “refresher” instead of full training was needed. For future studies, we recommend conducting assessments and training on separate days, to decrease the burdens on participants. Finally, some sessions were interrupted by a poor internet connection. In the present study, this problem was solved by reconnecting the computer to the internet via a mobile hotspot. As some rural regions do not have fast and reliable internet connections, future studies could provide additional solutions, such as Wi-Fi amplifiers or mobile Wi-Fi routers to overcome these problems.

### 4.3. Preliminary Efficacy of Intensive Speech-Language Telerehabilitation

This pilot feasibility trial also investigated the initial efficacy of the combined treatment programme, which was based on an established intervention programme for post-stroke aphasia (see [29] for details of the concept development phase). After ten sessions of naming training and ten sessions of communicative-pragmatic therapy, both participants showed considerable improvement in trained words and trained communicative-pragmatic subtests. Importantly, both types of treatment were delivered as teletherapy, with a trained speech and language therapist delivering the treatment via a video conferencing platform. Other studies have already demonstrated in small samples of pwPPA that SLT delivered as teletherapy is feasible for pwPPA and that it can be as effective as traditional in-person treatment [20,21,22,25]. For example, Dial and colleagues [20] demonstrated similar gains following in-person or remotely delivered lexical retrieval or script training in pwPPA. Similarly, Rogalski and colleagues [25] suggested that pwPPA were able to use a web-based teletherapy and that this approach improved functional communication and communication confidence of pwPPA. However, remote delivery of the specific treatment programme introduced in the current study had not yet been investigated. Thus, this is the first study to demonstrate that the tele-adaptation of both naming and communicative-pragmatic therapy induces a considerable training effect in pwPPA, thereby providing initial evidence for the efficacy of this new approach. Crucially, the combination of lexical naming with more interactive communicative-pragmatic approaches directly addressed treatment priorities that were identified as being particularly important for pwPPA and their caregivers [28,29].

Consistent with recent reviews of intervention studies in pwPPA [14,44,45,46,47], transfer to untrained items was not observed. This emphasises the importance of individualised and patient-relevant training item selection. It should also be noted that the type of naming treatment used in our study may not be sufficient to induce transfer in PPA, although such effects have been reported in post-stroke aphasia, albeit with a higher treatment dose (i.e., 30 hrs of naming therapy over two weeks) [30]. However, it is conceivable that the combination of tDCS with other treatment approaches that require more extensive engagement with specific semantic concepts (e.g., semantic feature analysis) may be suited to enhance transfer effects [46]. Moreover, the present case series focused on the feasibility of the overall intervention and not efficacy. A currently planned larger feasibility trial will increase naming therapy dose and also involve a long-term follow-up period, to investigate the maintenance of potential treatment effects and add-on benefits of tDCS on measures of transfer and generalisation.

Finally, we also observed considerable interindividual differences in performance levels during the baseline assessment and the degree of treatment success. Notably, baseline difficulty levels of untrained compared to trained items were lower in both patients and naming impairment was mild in one of the patients (i.e., resulting in near ceiling performance at baseline, thereby limiting the selection of “untrained items”). While both participants improved overall during communicative-pragmatic training, one of the participants exhibited a decline in performance in one of the treated subtests. Likely, this is explained by response variability expected in the aphasia population of all etiologies [48,49,50,51,52]. Furthermore, because of the pre-trial feasibility character of the present study, conclusions regarding efficacy are not permissible. Future studies with larger and more diverse samples are needed to investigate the efficacy of this combined treatment approach.

Also, tDCS was applied in a cross-over design, to be able to directly compare the sensations of both conditions in the same participant. Therefore, it was not possible to compare active versus sham tDCS. Although the overall level of perceived sensations differed considerably between the two pwPPA and was more pronounced in the sham condition in one pwPPA, none of them was able to indicate the order of stimulation, suggesting efficient blinding of the stimulation. However, future studies are needed to investigate the potentially beneficial add-on effect of tDCS on naming and communicative-pragmatic treatment.

### 4.4. Limitations

To the best of our knowledge, this pilot feasibility study is the first to combine two daily sessions of home-based tDCS with an intensive tele-SLT programme, focussed on the top priorities of pwPPA (naming and communication). There are several limitations to our approach. First, tDCS was applied in a cross-over design, as we were interested in within-participant tolerability of tDCS. A disadvantage of this approach is that no conclusions can be drawn about the additional effect of tDCS on speech and language performance. A future study would need to use a parallel group design, with one group receiving active tDCS and one group receiving sham tDCS to address this question. Moreover, the participants were already involved in the development of the intervention and therefore had considerable knowledge of the intervention and procedures prior to participation so that no separate training sessions were needed. Future studies need to take into account a more extensive training session to ensure the feasibility of such combined programmes. Another possible limitation is that both participants had been diagnosed with lvPPA for approximately one year. Hence, conclusions from this pilot study regarding feasibility and preliminary efficacy may not be transferable to people with other PPA variants (which may be associated with other comorbidities, such as motor problems in the nonfluent/agrammatic variant or behavioural symptoms in the semantic variant) or later stages of the disease. Finally, one investigator was present at the participants’ homes throughout the intervention phase. Although pwPPA and caregivers were instructed to carry out all preparatory actions independently and to minimise the interaction with the investigator, it cannot be ruled out that the mere presence of an on-site investigator could have influenced the participants. Also, while these findings are promising, they should be interpreted with caution due to the small sample size. Therefore, future studies need to establish feasibility without the presence of an on-site investigator and in a larger and more diverse sample of pwPPA.

## 5. Conclusions

In this case series, we describe the feasibility and tolerability of a novel intensive home-based tDCS and telerehabilitation programme, co-developed with pwPPA and caregivers. The telerehabilitation approach combines naming training with a communicative-pragmatic treatment, thereby targeting key treatment priorities of pwPPA. The present study is the first to demonstrate the feasibility, tolerability, safety and preliminary efficacy of a novel speech language teletherapy approach for pwPPA, combined with adjunct home-based tDCS. Our results demonstrate that this novel telerehabilitation intervention can be implemented successfully, which has the potential to enhance access to effective treatment for pwPPA. Future studies are needed to investigate the feasibility and efficacy of this novel treatment approach in a larger and more diverse sample.

## Figures and Tables

**Table 1:** Results: Communicative Pragmatic Abilities (KOPS).

Participant 1	Pretest (%)	Post-Test (%)
Word comprehension in context	100	100
*Referring to everyday objects*	70	43
Basic communicative actions	100	100
Conveying personal information	100	100
Conveying general information	83	97
*Complex communicative actions (individual actions)*	47	90
*Complex communicative actions (role-plays)*	45	85
Understanding/reconstructing directions	55	50
Generating directions	50	50
Total KOPS score (percent)	75	82
**Participant 2**	**Pretest (%)**	**Post-Test (%)**
Word comprehension in context	100	100
Referring to everyday objects	100	100
Basic communicative actions	100	100
Conveying personal information	100	100
Conveying general information	100	100
*Complex communicative actions (individual actions)*	77	100
*Complex communicative actions (role-plays)*	70	90
*Understanding/reconstructing directions*	90	100
*Generating directions*	75	100
Total KOPS score (percent)	92	99

Note: Subtests selected for treatment are shown in italics.

## Data Availability

Data are available from the corresponding author upon reasonable request. The data are not publicly available due to privacy or ethical restrictions.

## Abbreviations

The following abbreviations are used in this manuscript:

PPA	Primary progressive aphasia
pwPPA	People with primary progressive aphasia
SLT	Speech and language therapy
tDCS	Transcranial direct current stimulation
